# A pilot study on the efficacy of GPT-4 in providing orthopedic treatment recommendations from MRI reports

**DOI:** 10.1038/s41598-023-47500-2

**Published:** 2023-11-17

**Authors:** Daniel Truhn, Christian D. Weber, Benedikt J. Braun, Keno Bressem, Jakob N. Kather, Christiane Kuhl, Sven Nebelung

**Affiliations:** 1 Department of Diagnostic and Interventional Radiology, University Hospital RWTH Aachen, Pauwels Street 30, 52074 Aachen, Germany; 2 Department of Orthopaedics and Trauma Surgery, University Hospital RWTH Aachen, Aachen, Germany; 3 University Hospital Tuebingen on Behalf of the Eberhard-Karls-University Tuebingen, BG Hospital, Schnarrenbergstr. 95, Tübingen, Germany; 4 Department of Radiology, Charité – Universitätsmedizin Berlin, corporate member of Freie Universität Berlin and Humboldt-Universität zu Berlin, Hindenburgdamm 30, 12203 Berlin, Germany; 5 Else Kroener Fresenius Center for Digital Health, Technical University Dresden, Dresden, Germany; 6 Department of Medicine I, University Hospital Dresden, Dresden, Germany; 7 Department of Medicine III, University Hospital RWTH Aachen, Aachen, Germany; 8 Medical Oncology, National Center for Tumor Diseases (NCT), University Hospital Heidelberg, Heidelberg, Germany

**Keywords:** Diseases, Musculoskeletal system, Musculoskeletal system, Diagnosis, Medical imaging, Therapeutics

## Abstract

Large language models (LLMs) have shown potential in various applications, including clinical practice. However, their accuracy and utility in providing treatment recommendations for orthopedic conditions remain to be investigated. Thus, this pilot study aims to evaluate the validity of treatment recommendations generated by GPT-4 for common knee and shoulder orthopedic conditions using anonymized clinical MRI reports. A retrospective analysis was conducted using 20 anonymized clinical MRI reports, with varying severity and complexity. Treatment recommendations were elicited from GPT-4 and evaluated by two board-certified specialty-trained senior orthopedic surgeons. Their evaluation focused on semiquantitative gradings of accuracy and clinical utility and potential limitations of the LLM-generated recommendations. GPT-4 provided treatment recommendations for 20 patients (mean age, 50 years ± 19 [standard deviation]; 12 men) with acute and chronic knee and shoulder conditions. The LLM produced largely accurate and clinically useful recommendations. However, limited awareness of a patient’s overall situation, a tendency to incorrectly appreciate treatment urgency, and largely schematic and unspecific treatment recommendations were observed and may reduce its clinical usefulness. In conclusion, LLM-based treatment recommendations are largely adequate and not prone to ‘hallucinations’, yet inadequate in particular situations. Critical guidance by healthcare professionals is obligatory, and independent use by patients is discouraged, given the dependency on precise data input.

## Introduction

Large language models (LLMs) have recently spread into virtually all aspects of life, including medicine. Within the first two months of its launch, chatGPT, the most popular LLM and the archetype of dialogue-based artificial intelligence, attracted more than 100 million users and, in 2023, averaged more than 13 million daily visitors^1,2^.

While chatGPT (based on the GPT-3.5-model) performed at or near the passing threshold of 60% accuracy when undergoing the three standardized examinations of the United States Medical Licensing Exam (USMLE)^3^, its successor GPT-4 as the latest state-of-the-art LLM performed considerably better^4^. In addition to exceeding the USMLE passing threshold by over 20 percentage points, GPT-4 performs medical reasoning similarly to well-studied experts^5^. Beyond taking tests, chatGPT has diagnostic and triage abilities close to practicing physicians when dealing with case vignettes of conditions with variable severity^6,7^. GPT-4 has a clinical value in diagnosing challenging geriatric patients^8^. It also generates broadly appropriate recommendations for common questions about cardiovascular disease prevention^9^, breast cancer prevention, and screening^10^. It can also generate structured radiologic reports from written (prosaic) text^11^, and draft Impressions sections of radiologic reports, even though the GPT-4-generated Impressions are not (yet) as good as the radiologist-generated ones regarding coherence, comprehensiveness, and factual consistency^12^. For a comprehensive overview of LLMs and their utilization in radiology, the reader is referred to recent review articles^13–15^.

Despite the growing popularity of LLMs, concerns have arisen regarding the validity and reliability of their recommendations, particularly in medicine. LLMs tend to produce convincing but factual incorrect text (commonly referred to as “hallucinations”), which raises the question if LLMs are sufficiently sophisticated to be used as resources for health advice or may pose a potential danger^16^. In particular, patients may rely on information provided by artificial intelligence without consulting healthcare professionals^17,18^.

Likely, LLMs will also be extensively used by radiologists in the future. In this era of complex interdisciplinary patient management, a radiologist’s work often does not end with submitting a report of an imaging study. Addressing patients’ concerns and questions and communicating appropriately with non-radiologist colleagues requires solid knowledge of treatment options, prioritization, and limitations.

Consequently, the present study aims to investigate the validity of treatment recommendations provided by GPT-4, explicitly focusing on common orthopedic conditions, where accurate diagnosis and appropriate treatment are crucial for patients' recovery and long-term well-being^19–21^. By analyzing the treatment recommendations derived from clinical MRI reports, we evaluate whether the advice given by GPT-4 is scientifically sound and clinically safe. We hypothesize that GPT-4 produces largely accurate treatment recommendations yet is at substantial risk of hallucinations and may thus pose a potential risk for patients seeking health advice.

## Materials and methods

### Study design and dataset characteristics

The local ethical committee (Medical Faculty, RWTH Aachen University, Aachen, Germany, reference number 23/111) approved this retrospective study on anonymized data and waived the requirement to obtain individual informed consent. All methods were carried out in accordance with relevant guidelines and regulations. Following local data protection regulations, the board-certified senior musculoskeletal radiologist with ten years of experience (SN) screened all knee and shoulder MRI studies and associated clinical reports produced during the clinical routine at our tertiary academic medical center (University Hospital Aachen, Aachen, Germany) during February and March of 2023. Ninety-four knee MRI studies and 38 shoulder MRI studies were available for selection. We selected ten studies per joint, ensuring various conditions with variable severity and complexity. Table 1 provides a synopsis of the selected imaging studies with patient demographics, referring disciplines, reasons for the exam, principal diagnoses and treatment recommendations, and a statement on whether the treatment recommendations were considered problematic. Supplementary Table 1 provides more details on the reported diagnoses. Intentionally, we included MRI reports from patients with different demographic characteristics (i.e., age and sex) and referrals from various clinical disciplines. The diagnosis was checked for coherence and consistency using the associated clinical documentation (e.g., history and physical findings) and other non-imaging findings (e.g., laboratory values, intra-operative findings, functional tests, and others). Consequently, MRI studies were disregarded if additional findings were incoherent, inconsistent, or contradictory with the reference diagnosis.

**Table 1:** Synopsis of demographics, referring departments, patient history, and principal diagnoses and treatment recommendations as well as a brief statement on whether the treatment recommendations were considered problematic. Abbreviations: AC—acromioclavicular, ACL—anterior cruciate ligament, ALPSA—anterior labroligamentous periosteal sleeve avulsion of the labrum, ER—emergency department, GH—glenohumeral, GLAD—glenolabral articular disruption, GP—general practitioner, HAGL—humeral avulsion of the glenohumeral ligament, INT—Internal Medicine, ISP—infraspinatus, OTS—orthopedic and trauma surgery, PCL—posterior cruciate ligament, PED – Pediatrics, PS—Plastic Surgery, RD—Referring department, SSC—subscapularis, SSP – supraspinatus. Supplementary Tables 1 and 2 provide further details on the reported diagnoses and treatment recommendations, respectively.

MRI Study		Age	Sex	Referral	Reason for exam	Principal reported diagnoses	Principal treatment recommendation	Treatment recommendation(s) problematic?
Knee	1	75	Male	INT	Undulating fever and swollen and painful knee	Joint infection with concomitant osteomyelitis and advanced degeneration	Address infection and inflammation (antibiotics) after blood tests	Yes
	2	68	Male	OTS	Worsening symptoms. Medial osteoarthritis	Medial compartmental osteoarthritis with bone changes and meniscus degeneration and chondropathy elsewhere in the joint	Conservative measures. If failing, surgical measures	No
	3	62	Male	OTS	Medial pain since fall several weeks ago	Insufficiency fracture of the medial femoral condyle and cartilage intact	Rest and avoid weight-bearing. Use crutches or cane	Yes
	4	62	Male	GP	Clinical suspicion of medial meniscus lesion	Horizontal posterior root tear of the medial meniscus	Conservative measures. If failing, surgical measures	Yes
	5	52	Female	ED	Joint pain since fall	Medial meniscus tear of posterior horn	Conservative measures. If failing, surgical measures	No
	6	48	Male	OTS	S/p knee dislocation. After reduction	Rupture of the ACL, medial collateral ligament, bucket handle tear of the medial meniscus (O'Donoghue triad), and rupture of the PCL, medial retinaculum, and popliteus muscle	Surgery. Conservative measures while waiting for surgery	Yes
	7	31	Female	OTS	Clinical suspicion of ACL or meniscus damage after skiing accident	Partial rupture/overstretching of the proximal ACL and the MCL. Tibial bone bruise	Conservative measures	No
	8	25	Female	OTS	Radiographic suspicion of bony avulsion of ACL	Bony avulsion of the ACL, impression fracture of the tibial plateau, and radial tear of the lateral meniscus posterior horn	Surgery	Yes
	9	14	Male	PED	S/p ACL reconstruction. Pain after knee distortion during fall	Partial ACL graft rupture and s/p lateral patellar dislocation	Conservative measures	Yes
	10	14	Female	PED	Regular follow-up	Constant osteochondroma of the distal femur (cartilage cap: 0.5 cm)	No treatment if asymptomatic. Surgical removal if symptomatic	Yes
Shoulder	1	68	Female	OTS	Palpable mass over shoulder. No pain	Subcutaneous lipoma and advanced attritive changes of the glenohumeral joint (rheumatic disease)	Conservative measures	Yes
	2	68	Female	GP	Impingement syndrome	Degenerative changes of the GH and AC joints. Partial ruptures of the SSP and SSC tendons	Conservative measures. If failing, surgical measures	Yes
	3	63	Male	OTS	Clinical suspicion of calcific tendinitis. Biceps pathology?	Calcific tendinitis and ISP tendinopathy	Conservative measures	No
	4	61	Male	OTS	S/p second dislocation	Hill-Sachs lesion, glenoid bone loss of 8%, HAGL and GLAD lesions	Conservative measures. If failing, surgical measures	No
	5	60	Male	OTS	Traumatic injury three months ago with supraspinatus tendon tear on ultrasound	Massive rotator cuff tear involving SSP, SSC, and ISP tendons with volume atrophy and fatty infiltration, as well as activated AC joint arthritis	Conservative measures. If failing, surgical measures	No
	6	60	Male	OTS	Acute-on-chronic shoulder pain. Post-instability osteoarthritis	Advanced osteoarthritis of GH and AC joints with SSP and ISP tendinopathy	Conservative measures. If failing, surgical measures	No
	7	34	Male	GP	S/p second dislocation	Hill-Sachs lesion, glenoid bone loss of 15%, ALPSA and GLAD lesions	Conservative measures. If failing, surgical measures	No
	8	47	Female	GP	Clinical suspicion of adhesive capsulitis	Adhesive capsulitis	Conservative measures. If failing, surgical measures	No
	9	32	Male	PS	Palpable swelling over the shoulder. No pain	No pathology, just a strong muscular build	Conservative measures	No
	10	53	Female	INT	Impingement syndrome for six months. Loss of abduction strength	Intratendinous lesion of SSP tendon with bursitis	Conservative measures. If failing, surgical measures	No

The selected MRI reports were extracted from the local Picture Archiving and Communication System (iSite, Philips Healthcare, Best, Netherlands) as intended for clinical communication, i.e., in German. The MRI reports were anonymized by removing the patient's name, age, sex, and reference to earlier imaging studies. In the history and reason-for-exam section, any reference that may influence treatment recommendations, e.g., “preoperative evaluation [requested]”, was removed, too.

### GPT-4 Encoding and Prompting

GPT-4 was accessed online (https://chat.openai.com/) on April 11th and 12th, 2023, and operated as the chatGPT March 23 version. Prompts were provided in a standardized format and the following sequence:

- Prompt #1: Please translate the following MRI report into English.
- Prompt #2: This is the MRI report of a [numerical age]-year-old [sex, woman/man]. Do the conditions need to be treated? And if so, how? Please be as specific as possible.
- Prompt #3: This is too unspecific. Please advise the patient on what to do. Imagine you are the treating physician. Prioritize your treatment recommendations—begin with what is most sensible and relevant.

Consequently, GPT-4 was provided with the patient’s age and sex only. The translated (English) version of the clinical MRI report was checked for overall quality and in terms of accuracy, consistency, fluency, and context by the senior musculoskeletal radiologist (SN), who holds the certificate of the Educational Commission for Foreign Medical Graduates (ECFMG). A new chat session was started for each patient to avoid memory retention bias.

Alongside the MRI reports, the treatment recommendations made by GPT-4 following the initial (prompt #2) and the follow-up request (prompt #3) were saved.

**Figure 1:**
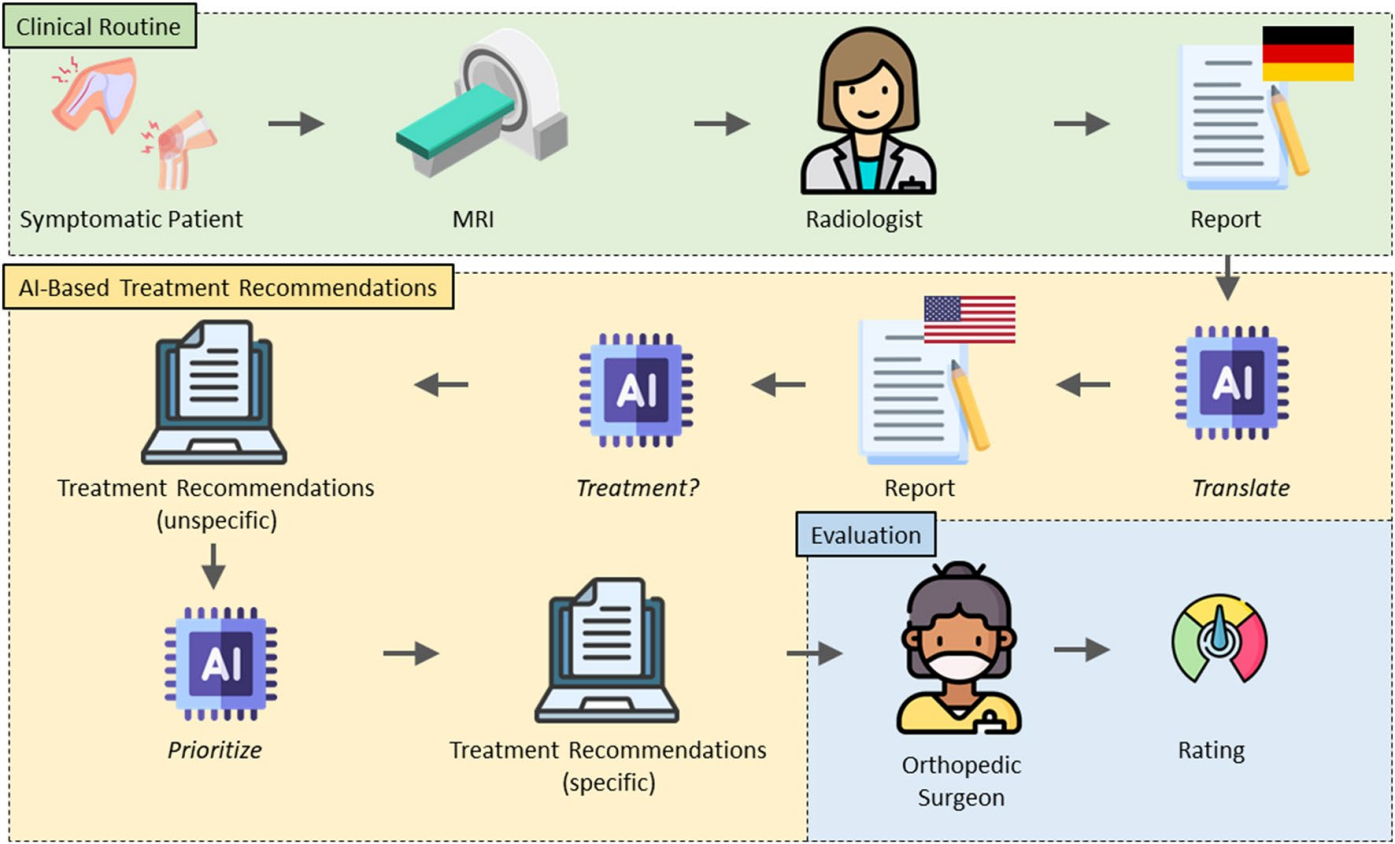
Workflow of the Artificial Intelligence-powered MRI-to-treatment recommendation pipeline. GPT-4, denoted as the AI icon, was prompted three times to translate the MRI report, provide general treatment recommendations, and prioritize its recommendations. Two experienced orthopedic surgeons rated the patient-specific treatment recommendations.

Figure 1 provides an overview of the workflow.

### Evaluation of treatment recommendations

Two board-certified and specialty-trained senior orthopedic surgeons with ten (BB) and 12 (CW) years of clinical experience in orthopedic and trauma surgery, evaluated the treatment recommendations made by GPT-4.

Both raters evaluated the treatment recommendations separately by answering the itemized questions in Table 2. Treatment recommendations were rated on Likert scales extending from 1 (*poor* or *strongly disagree*) to 5 (*excellent* or *strongly agree*) regarding overall quality, scientific and clinical basis, and clinical usefulness and relevance. Whether the treatment recommendations are up-to-date and consistent was rated on a binary basis, i.e., yes or no.

**Table 2:** Itemized questions used to rate the treatment recommendations for each MRI report. Two experienced orthopedic surgeons used Likert scales (1 to 5) or binary schemes (yes or no). Additionally, raters were asked to provide (free-text) comments for each patient.

Question to evaluate	Possible answers
The overall quality of the treatment recommendations is	Poor [1]—Fair [2]—Good [3]—Very good [4]—Excellent [5]
Treatment recommendations are based on scientific and clinical evidence	Strongly disagree [1]–Disagree [2]—Neutral [3]—Agree [4]—Strongly Agree [5]
Treatment recommendations are clinically useful and relevant	Strongly disagree [1]—Disagree [2]—Neutral [3]—Agree [4]—Strongly Agree [5]
Treatment recommendations are up to date	Yes–no
Treatment recommendations are consistent	Yes–no

Afterward, both raters held a consensus meeting where discrepant ratings were discussed until a consensus was reached. Only the consented scores were registered and subsequently analyzed.

## Results

In all responses, GPT-4 consistently disclaimed it was not a doctor. GPT-4 offered some general information on the conditions and potential treatment options, yet would not be willing to provide specific medical advice. It repetitiously stressed the importance of consulting with healthcare professionals for personalized treatment recommendations.

GPT-4 explained the MRI findings separately using layman’s language. It continuously worked down the list of findings when formulating its treatment recommendations, following the radiologist’s prioritization.

**Figure 2:**
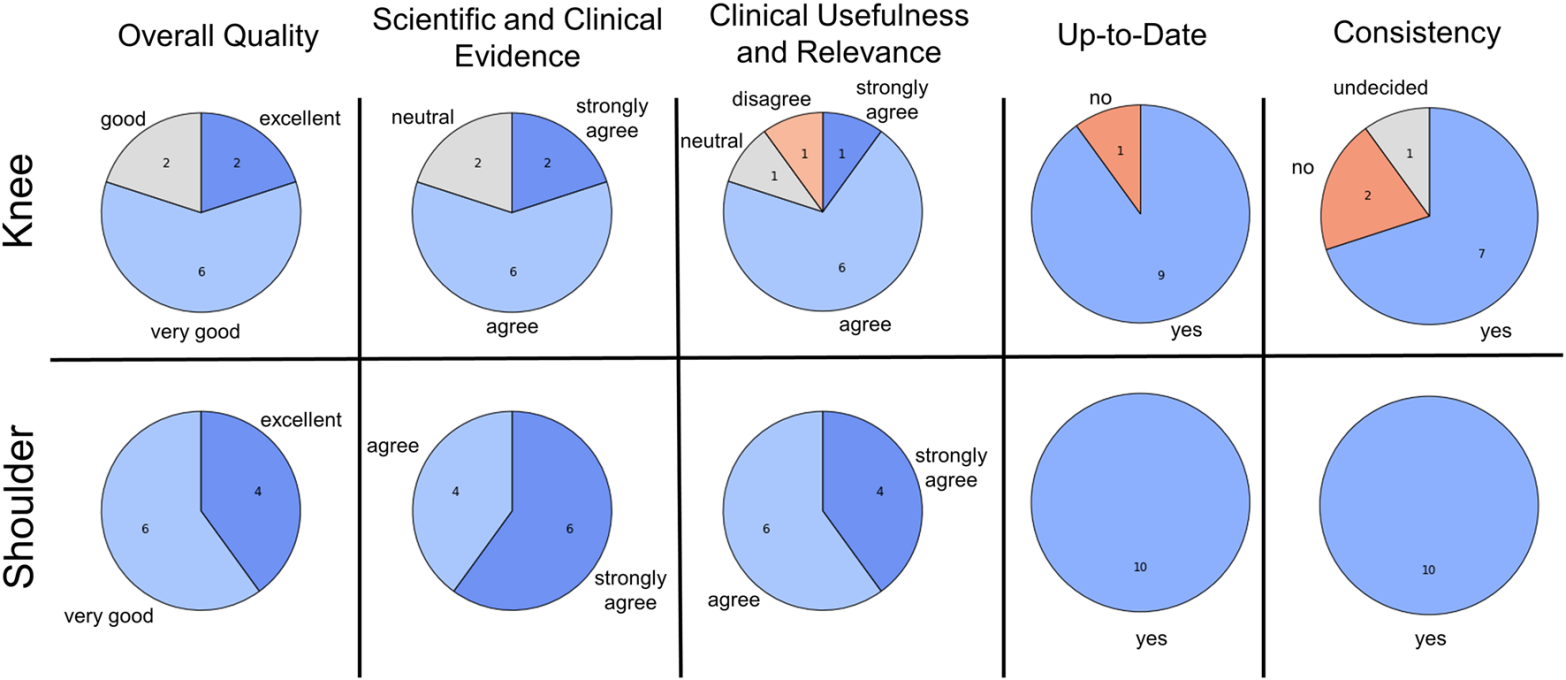
Multidimensional ratings of the treatment recommendations provided by GPT-4. In a consensus meeting, two experienced orthopedic surgeons evaluated the treatment recommendations for various knee and shoulder conditions derived from clinical MRI reports. Ratings were based on five-item Likert scales, and counts were provided only for selected answers.

The overall quality of the treatment recommendations was rated as good or better for the knee and shoulder. Similarly, the recommendations were mainly up-to-date and consistent, adhering to clinical and scientific evidence and clinically useful/relevant (Fig. 2). Notably, the treatment recommendations provided for the shoulder were rated more favorably. We did not find signs of hallucinations, i.e., seemingly correct responses that (i) were non-sensical when considered against common knowledge in radiology or orthopedic surgery/traumatology or (ii) inconsistent with framework information or conditions stated in the radiologist’s request. Moreover, we did not find signs of speculations or oversimplifications.

**Figure 3:**
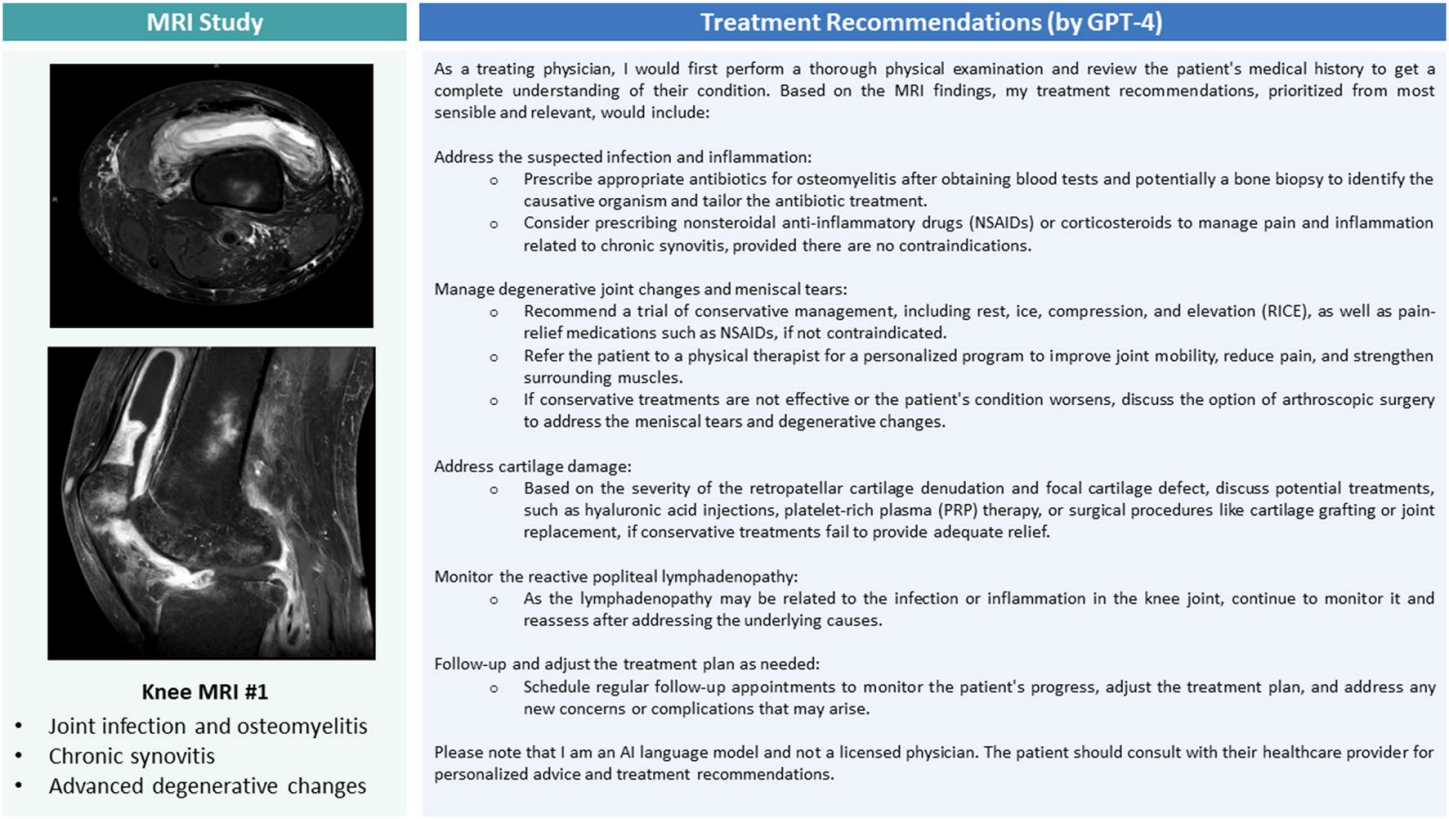
Representative knee joint MR images of a patient with a joint infection, key MRI report findings, and specific treatment recommendations by GPT-4. Axial proton density-weighted fat-saturated image above the patella (upper image) and sagittal post-contrast T1-weighted fat-saturated image through the central femur diaphysis (lower image). Of all 20 MRI studies/reports and associated treatment recommendations, these treatment recommendations were rated lowest.

**Figure 4:**
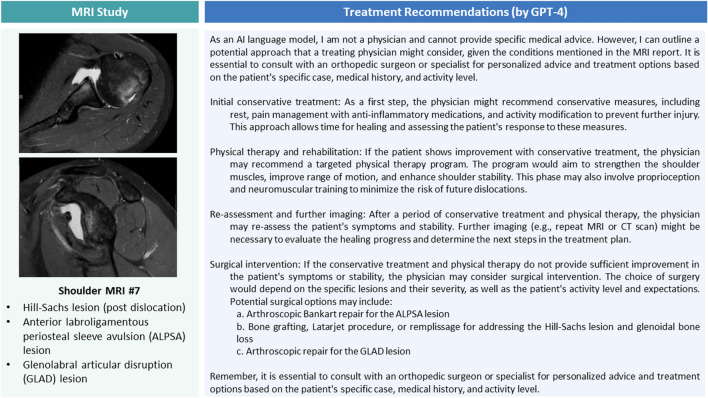
Representative shoulder joint MR images of a patient after re-dislocation, key MRI report findings, and specific treatment recommendations by GPT-4. Axial and parasagittal proton density-weighted fat-saturated images through the humeral head and glenoid, respectively.

GPT-4's treatment recommendations generally followed a schematic approach. In most cases, conservative treatment was recommended initially, regularly accompanied by physical therapy. Surgical treatment was considered a potential option for those patients where conservative treatment, including physical therapy, would not yield satisfactory results. Representative MR images, MRI report findings, and GPT-4-based treatment recommendations are provided for the knee (Fig. 3) and shoulder (Fig. 4).

The two orthopedic surgeons agreed that some recommendations could have been more specific. In numerous patients, GPT-4 provided general advice instead of tailoring the treatment recommendations to the particular condition or patient, thereby limiting their clinical usefulness. Furthermore, GPT-4 tended to err on the side of caution, recommending more conservative treatment options and leaving the decision for surgery to the specialists to be consulted. Supplementary Table 2 provides further details on the treatment recommendations for each patient/MRI report and associated comments by the two orthopedic surgeons.

## Discussion

Our study suggests that GPT-4 can produce valuable treatment recommendations for common knee and shoulder conditions. The recommendations were largely up-to-date, consistent, clinically useful/relevant, and aligned with the most recent clinical and scientific evidence.

We observed signs of reasoning and inference across multiple key findings. For example, GPT-4 correctly deduced that meniscus tears may be associated with bone marrow edema (as a sign of excessive load transmission). Hence, its recommendation to “address focal bone marrow edema: As this issue could be related to the medial meniscus tear […]" was entirely plausible.

Similarly, GPT-4 demonstrated considerable foresight as it recommended organizing post-surgical care and rehabilitation for the patient with multi-ligament knee injuries and imminent surgery. Whether this recommendation can be regarded as "planning" is questionable, though, as true planning abilities in the non-medical domain are still limited^4,16^. Instead, these recommendations are likely based on the schematic treatment regime that GPT-4 encountered in its training data.

Interestingly, GPT-4 recommended lifestyle modifications, i.e., weight loss and low-impact exercise, and assistive devices (such as braces, canes, or walkers) for shoulder degeneration. While these are sensible and appropriate recommendations for knee osteoarthritis, such recommendations are of doubtful value in shoulder osteoarthritis. In patients with shoulder osteoarthritis or degeneration, exercises to improve the range of motion were not recommended, even though they are indicated^22^. Again, this observation is likely attributable to the statistical modeling behavior of GPT-4, given the epidemiologic dominance of knee OA over shoulder OA.

Additional limitations of GPT-4 became apparent when the model was tasked to make treatment recommendations for patients with complex conditions or multiple relevant findings.

Critically, the patient with septic arthritis of the knee was not recommended to seek immediate treatment. This particular treatment recommendation, or rather the failure to stress its urgency, is negligent and dangerous. Septic arthritis constitutes a medical emergency, which may lead to irreversible joint destruction, morbidity, and mortality. Literature studies report mortality rates of 4% to 42%^23–25^. Furthermore, because of the stated cartilage damage in this patient, GPT-4 also recommended cartilage resurfacing treatment. However, doing so in a septic joint is contraindicated and medical malpractice^26^.

GPT-4 was similarly unaware of the patient’s overall situation after knee dislocation. Even though the surgical treatment recommendations for multi-ligament knee injuries were plausible, a potential concomitant popliteal artery injury was not mentioned. It occurs in around 10% of knee dislocations and may dramatically alter treatment^2^.

Remarkably, we did not find signs of so-called "hallucinations", i.e., GPT-4 "inventing" facts and confidently stating them. Even though speculative at this stage, the absence of such hallucinations may be due to the substantial and highly specific information provided in the prompt (i.e., the entire MRI report per patient) and our straightforward prompting strategy compared to more suggestive promptings of other studies^16^.

No patient is treated on the basis of the MR images or the MRI report. Nonetheless, using real-patient (anonymized) MRI reports rather than artificial data, increases our study’s applicability and impact.

However, while GPT-4 offered treatment recommendations, it is crucial to understand that it is not a replacement for professional medical evaluation and management. The accuracy of its recommendations is largely contingent upon the input's specificity, correctness, and reasoning, which is typically not how a patient would phrase the input and prompt the tool. Therefore, LLMs, including GPT-4, should be used as supplementary resources by healthcare professionals only, as they provide critical oversight and contextual judgment. Optimally, healthcare professionals know a patient's constitution and circumstances to provide effective, safe, and nuanced diagnostic and treatment decisions. Consequently, we caution against the use of GPT-4 by laypersons for specific treatment suggestions.

Along similar lines, integrating LLMs into clinical practice warrants ethical considerations, particularly regarding medical errors. First and foremost, their use does not obviate the need for professional judgment from healthcare professionals who are ultimately responsible for interpreting the LLM's output. As with any tool applied in the clinic, LLMs should only assist (rather than replace) healthcare professionals. However, the safe and efficient application of LLMs requires a thorough understanding of their capabilities and limitations. Second, developers must ensure that their LLMs are rigorously tested and validated for clinical use and that potential limitations and errors are communicated, necessitating ongoing performance monitoring. Third, healthcare institutions integrating LLMs into their clinical workflows should establish governance structures and procedures to monitor performance and manage errors. Fourth, the patient (as a potential end-user) must be made aware of the potential for hallucinations and erroneous and potentially harmful advice. Our study highlights the not-so-theoretical occurrence of harmful advice—in that case, we advocate a framework of shared responsibility. The healthcare professional is immediately responsible for patient care if involved in alleged malpractice. Simultaneously, LLM developers and healthcare institutions share an ethical obligation to maximize the benefits of LLMs in medicine while minimizing the potential for harm. While there is no absolute safeguard against medical errors, informed patients make informed decisions—this applies to LLMs as to any other health resource utilized by patients seeking medical advice.

Importantly, LLMs, including GPT-4, are currently not approved as medical devices by regulatory bodies. Therefore, LLMs cannot and should not be used in the clinical routine. However, our study indicates that the capability of LLMs to make complex treatment recommendations should be considered in their regulation.

Moreover, the recent advent of multimodal LLMs such as GPT-4Vision (GPT-4V) has highlighted the (potentially) vast capacities of multimodal LLMs in medicine. In practice, the text prompt (e.g., original MRI report) could be supplemented by select MR images or additional clinical parameters such as laboratory values. Recent literature evidence studying patients in intensive care confirmed that models trained on imaging and non-imaging data outperformed their counterparts trained on only one data type.^27^ Consequently, future studies are needed to elucidate the potentially enhanced diagnostic performance as well as the concomitant therapeutic implications.

When evaluating the original MRI report (in German) and its translated version (in English), we observed them to be excellently aligned regarding accuracy, consistency, fluency, and context. This finding is confirmed by earlier literature, indicating an excellent quality of GPT-4-based translations, at least for high-resource European languages such as English and German^28^. Inconsistent taxonomies in MRI reports may be problematic for various natural language processing tasks but did not affect the quality of report translations in this study.

Our study has limitations. First, we studied only a few patients, i.e., ten patients each for the shoulder and knee. Consequently, our investigation is a pilot study with preliminary results and lacks a solid quantitative basis for statistical analyses. Consequently, no statistical analysis was attempted based on our dataset. Second, to enhance its depth and relevance to clinical scenarios, GPT-4’s predictions need to be more specific. Additional ‘fine-tuning’ and domain-specific training using medical datasets, clinical examples, and multimodal data may enhance its robustness and specificity as well as its overall value as a supplementary resource in healthcare. Third, the patient spectrum was broad. A more thorough performance assessment would require substantially more patients with rare conditions and subtle findings to be included. Fourth, treatment recommendations were qualitatively judged by two experienced orthopedic surgeons. Given the excellent level of inter-surgeon agreement, we consider the involvement of two surgeons sufficient, yet involving three or more surgeons could have strengthened the outcome basis even further. Fifth, the tendency of GPT-4 to give generic and unspecific answers and to err on the side of caution rendered it challenging to assess its adherence to guidelines or best practices exactly. Sixth, we used a standardized and straightforward way of prompting GPT-4. After more extensive modifications of these prompts, outcomes may be different.

In summary, common conditions and associated treatment recommendations were well handled by GPT-4, whereas the quality of the treatment recommendations for rare and more complex conditions remains to be studied. Most treatment recommendations provided by GPT-4 were largely consistent with the expectations of the evaluating orthopedic surgeons. The schematic approach used by GPT-4 often aligns well with the typical treatment progression in orthopedic surgery and sports medicine, where conservative treatments are usually attempted first, and surgical intervention is considered only after the failure of conservative treatments.

## Conclusion

In conclusion, GPT-4 demonstrates the potential to provide largely accurate and clinically useful treatment recommendations for common orthopedic knee and shoulder conditions. Expert surgeons rated the recommendations at least as "good", but the patient's situation and treatment urgency were not fully considered. Therefore, patients need to consult healthcare professionals for personalized treatment recommendations, while GPT -4 may be a supplementary resource rather than a replacement for professional medical advice after regulatory approval.

### Supplementary Information

Supplementary Information.

## Data Availability

Data generated or analyzed during the study (i.e., the original MRI reports) are available from the corresponding author upon reasonable request.

## Abbreviations

AI: Artificial intelligence
LLM: Large language model
MRI: Magnetic resonance imaging
