# Author Correction: A pilot study on the efficacy of GPT-4 in providing orthopedic treatment recommendations from MRI reports

**DOI:** 10.1038/s41598-024-56029-x

**Published:** 2024-03-05

**Authors:** Daniel Truhn, Christian D. Weber, Benedikt J. Braun, Keno Bressem, Jakob N. Kather, Christiane Kuhl, Sven Nebelung

**Affiliations:** 1 Department of Diagnostic and Interventional Radiology, University Hospital RWTH Aachen, Pauwels Street 30, 52074 Aachen, Germany; 2 Department of Orthopaedics and Trauma Surgery, University Hospital RWTH Aachen, Aachen, Germany; 3 University Hospital Tuebingen on Behalf of the Eberhard-Karls-University Tuebingen, BG Hospital, Schnarrenbergstr. 95, Tübingen, Germany; 4 Department of Radiology, Charité – Universitätsmedizin Berlin, corporate member of Freie Universität Berlin and Humboldt-Universität zu Berlin, Hindenburgdamm 30, 12203 Berlin, Germany; 5 Else Kroener Fresenius Center for Digital Health, Technical University Dresden, Dresden, Germany; 6 Department of Medicine I, University Hospital Dresden, Dresden, Germany; 7 Department of Medicine III, University Hospital RWTH Aachen, Aachen, Germany; 8 Medical Oncology, National Center for Tumor Diseases (NCT), University Hospital Heidelberg, Heidelberg, Germany

Correction to: *Scientific Reports* 10.1038/s41598-023-47500-2, published online 17 November 2023

The Author Contributions section in the original version of this Article was incomplete.

“Guarantors of integrity of entire study, all authors; study concepts/study design or data acquisition or data analysis/interpretation, all authors; manuscript drafting or manuscript revision for important intellectual content, all authors; approval of final version of submitted manuscript, all authors; literature research, D.T., S.N.; experimental studies, D.T., C.K., C.D.W., B.J.B., S.N., D.T.; data analysis, D.T., S.N.; manuscript editing: all authors.”

now reads:

“Guarantors of integrity of entire study, all authors; study concepts/study design or data acquisition or data analysis/interpretation, all authors; manuscript drafting, D.T.; manuscript revision for important intellectual content, all authors; approval of final version of submitted manuscript, all authors; literature research, D.T., S.N.; experimental studies, D.T., C.K., C.D.W., B.J.B., S.N., D.T.; data analysis, D.T., S.N.; manuscript editing: all authors.”

The original Article has been corrected.

