# Supplementary material for: A pilot study on the efficacy of GPT-4 in providing orthopedic treatment recommendations from MRI reports
Source: Sci Rep. 2023 Nov 17;13:20159. doi: 10.1038/s41598-023-47500-2 (PMC10656559; doi:10.1038/s41598-023-47500-2)
Supplement: Supplementary file 1 — Supplementary Information. [file 41598_2023_47500_MOESM1_ESM.pdf]

Supplementary Material

Title: A Pilot Study on the Efficacy of GPT-4 in Providing Orthopedic Treatment Recommendations from MRI Reports

Journal: Scientific Reports

Supplementary Tables

Supplementary Table 1: Details of reported diagnoses as indicated in the respective MRI reports. For comprehensibility and clarity, the key findings of each MRI report were summarized into two principal diagnoses. MRI studies and abbreviations as defined in **Table 1**.

MRI Study		Details of Reported Diagnoses
**Knee**	1	1. Joint infection with extensive effusion, chronic synovitis, and concomitant osteomyelitis of the femur, tibia, and patella.<br>2. Signs of advanced degeneration with meniscal extrusion, lateral meniscus tear, eburnation of retropatellar cartilage, and focal cartilage defects of the lateral tibia.
	2	1. Medial compartmental osteoarthritis with bone changes and medial meniscus degeneration.<br>2. Partial-thickness cartilage defects with subchondral bone marrow edema in the lateral trochlear facet. Retropatellar chondropathy.
	3	1. Insufficiency fracture of the medial femoral condyle. No extension into the joint. Adjacent cartilage intact.<br>2. Mucoid degeneration of the medial meniscus (posterior horn) without a tear.
	4	1. Horizontal posterior root tear of the medial meniscus.<br>2. Mild mucoid degeneration of the ACL and PCL.
	5	1. Medial meniscus tear (posterior horn).<br>2. Infrapatellar hematoma, likely traumatic.
	6	1. Rupture of the ACL, medial collateral ligament, and bucket handle tear of the medial meniscus (O'Donoghue triad).<br>2. Accompanying rupture of the posterior cruciate ligament, medial retinaculum, and popliteus muscle.
	7	1. Partial rupture/overstretching of the proximal ACL. Bone bruise in the dorsolateral tibial plateau.<br>2. Partial rupture of the medial collateral ligament.
	8	1. Bony avulsion of the ACL and impression fracture of the tibial plateau (step of 1 mm).<br>2. Radial tear of the lateral meniscus posterior horn.
	9	1. Partial ACL graft rupture.<br>2. Findings consistent with s/p lateral patellar dislocation, i.e., contusion edema of the medial patella and lateral distal femur, bony avulsion of the medial patellofemoral ligament.
	10	1. Constant manifestation of osteochondroma (cartilaginous exostosis) of the distal femur; cartilage cap of 0.5 cm thickness.

**Shoulder**	1	1. Subcutaneous lipoma.<br>2. Advanced attritive changes of the glenohumeral joint and cystic changes in the humerus, suggesting rheumatic origin.
	2	1. Degenerative changes of the glenohumeral joint and activated arthritis of the acromioclavicular joint with narrowing of the subacromial space and bursitis.<br>2. Partial rupture of the supraspinatus and subscapularis tendons.
	3	1. Calcific tendinitis and tendinopathy of the infraspinatus.
	4	1. Findings consistent with acute shoulder re-dislocation, i.e., Hill-Sachs lesion and glenoid bone loss of 8% (on-track shoulder), humeral avulsion of the glenohumeral ligament (HAGL), and glenolabral articular disruption (GLAD).<br>2. Partial-thickness articular side lesion of the supraspinatus tendon.
	5	1. Massive rotator cuff tear involving the supraspinatus, infraspinatus, and subscapularis tendons with significant volume atrophy, fatty infiltration, edema of the muscles, and secondary cranialization of the humerus.<br>2. Activated AC joint arthritis.
	6	1. Advanced glenohumeral osteoarthritis with deformation of the humerus and cartilage loss. Advanced acromioclavicular joint arthritis without subacromial narrowing. Supraspinatus and infraspinatus tendinopathy.<br>2. Joint effusion and secondary synovial osteochondromatosis.
	7	1. Findings consistent with acute shoulder re-dislocation, i.e., Hill-Sachs lesion and glenoid bone loss of 15% (off-track shoulder), anterior labroligamentous periosteal sleeve avulsion (ALPSA) of the labrum, and glenolabral articular disruption (GLAD).
	8	1. Adhesive capsulitis.<br>2. Suspected subacromial narrowing with supraspinatus tendinopathy and subacromial and subcoracoid bursitis.
	9	1. Strong muscular build with prominent pectoralis muscle as the correlate of the palpable swelling. No mass. No edema.
	10	1. Tendinopathy and intratendinous lesion of the supraspinatus tendon.<br>2. Subcoracoid and and subacromial bursitis.

Supplementary Table 2: Detailed treatment recommendations by GPT-4 for each patient/MRI report and associated comments by the two experienced orthopedic surgeons. Both orthopedic surgeons evaluated and commented on the positive and negative aspects of the treatment recommendations. For comprehensibility and clarity, the treatment recommendations for each patient were summarized into three principal recommendations. The RICE protocol consists of rest, ice, compression, and elevation. MRI studies and abbreviations as defined in **Table 1**.

MRI Study		Treatment recommendations	Comment[s]
Knee	1	1. Address infection and inflammation (antibiotics) after blood tests. Consider bone biopsy.<br>2. Pain management as needed.<br>3. Manage conservatively degenerative joint changes, meniscal tears, and cartilage damage. If conservative treatment fails, consider arthroscopic surgery.	(i) Adequate weighting of conditions with acute infection as the primary (acute) problem and degeneration as a secondary problem.<br>(ii) Acute infection is misjudged in its urgency; consequently, the recommendation to see a doctor is given without specific timing. In this patient, fever requires urgent/emergency consultation as septic arthritis is associated with substantial morbidity and mortality if treated too late or inadequately (1).<br>(iii) Arthroscopic and cartilage resurfacing procedures discussed but highly problematic in acute infection and controversial in elderly patients (>75 years).
	2	1. Conservative measures include physical therapy, pain management, weight loss (if overweight), assistive devices (e.g., cane or brace), and activity modification.<br>2. Surgical options if conservative measures fail, i.e., arthroscopy, osteotomy, or partial/total knee replacement.	(i) Comprehensive list of conservative and surgical treatment options provided.<br>(ii) Further diagnostic work-up options for therapeutic decision-making, such as long leg radiographs, are not mentioned.
	3	1. Rest and avoid weight-bearing activities. Use crutches or a cane to assist with mobility. Apply ice. Manage pain. Gradual return to weight-bearing guided by a physical therapist is permitted once healing is underway.<br>2. Manage meniscus degeneration by monitoring the patient. Consider physical therapy if symptomatic. Consider arthroscopic surgery if conservative treatment fails.	(i) Reduced weight-bearing should be combined with short-term anticoagulant treatment, otherwise adequate treatment recommendations.
	4	1. Start with conservative treatment, i.e., RICE protocol and physical therapy. Consider arthroscopic surgery if conservative treatment fails.<br>2. Conservative management of degenerative cruciate ligaments, such as muscle strengthening, activity modification, and pain management.	(i) Arthroscopic refixation of the (partially) torn posterior root of the medial meniscus should be seriously considered, even in a 62-year-old patient. Factors relevant to surgical indication, i.e., biological age and athletic demand, are not mentioned.<br>(ii) Recommendations should be more specific. Patient-specific factors, such as overweight or leg axis, are disregarded but may affect treatment. Otherwise, adequate treatment recommendations.
	5	1. Address meniscus tear through conservative treatment, i.e., RICE protocol, pain management as needed, and physical therapy to strengthen muscles.<br>2. Consider arthroscopic surgery to remove or repair damaged meniscus tissue if conservative measures fail.<br>3. Manage infrapatellar hematoma conservatively, i.e., RICE protocol.	(i) Adequate treatment recommendations.
	6	1. Conservative management, i.e., RICE protocol, pain management, and physical therapy, is recommended for less severe injuries. In this patient, surgical treatment seems necessary due to the extent of the damage.	(i) Although surgery is unequivocally recommended due to the severity and complexity of injuries, knee dislocation is not addressed as their common cause. Consequently, checking the vascular status should have been

		2. Meanwhile (while waiting for surgery), consider additional immobilization (i.e., splint or brace).<br>3. Organize post-surgical care and rehabilitation.	recommended, as 10 % of knee dislocations are associated with popliteal artery injuries (2).<br>(Yet, a time-critical vascular injury may have been ruled out a priori because there was enough time for the MRI scan.)<br>(ii) Otherwise, adequate treatment recommendations.
	7	1. Conservative treatment of partial ligament injuries, i.e., RICE protocol, pain management, brace, and physical therapy.<br>2. Close monitoring to adjust the treatment plan and determine the timing of safe return to sports/other activities.	(i) Adequate treatment recommendations, yet too unspecific. Depending on the physical exam brace may be restricted, weight-bearing reduced, and short-term anticoagulant treatment initiated.
	8	1. Bony avulsion of the ACL should be addressed by surgical ACL reconstruction using a graft.<br>2. Radial tear of the lateral meniscus posterior horn should be addressed arthroscopically to repair or remove the damaged portion.<br>3. Manage the tibial plateau impression fracture, either conservatively by immobilization, pain management, and physical therapy or surgically if the fracture is severe or associated with instability.	(i) Bony avulsions of the ACL should be treated by direct surgical refixation of the avulsed ligament, not by graft-based ligament reconstruction.<br>(ii) Otherwise, adequate treatment recommendations.
	9	1. Address partial rupture of the ACL graft conservatively, i.e., through physical therapy, bracing, and activity modification. If the knee remains unstable, consider arthroscopic repair or revision of the ACL reconstruction.<br>2. Address patellar dislocation by reduction of the joint, immobilization in a brace, and anti-inflammatory medication. Physical therapy to strengthen quadriceps and hamstring muscles and improve knee stability. If there are recurrent dislocations, consider surgical repair or reconstruction of the medial patellofemoral ligament.	(i) The paradigm of conservative-only treatment after 1st-time patellar dislocations has shifted. At-risk patients for recurrent instability should be considered for early surgical intervention (3). Instability scoring systems not mentioned.<br>(ii) Otherwise, adequate treatment recommendations.
	10	1. If asymptomatic, osteochondroma does not require treatment. If symptomatic (e.g., pain, nerve compression, or restricted joint movement), surgical removal may be considered.<br>2. Regular follow-up imaging studies (MRI, radiography) are recommended.	(i) The potential of malignant transformation to chondrosarcoma (~1% in solitary osteochondromas) (4) is not discussed.
**Shoulder**	1	1. Manage lipoma conservatively unless associated with pain, functional impairment, or cosmetic concern. In those cases, consider surgical excision.<br>2. Treat degenerative/rheumatic joint changes through pain management, physical therapy, corticosteroid/hyaluronic acid injections, lifestyle changes (weight loss, low-impact exercise), and assistive devices such as braces, canes, or walkers.	(i) Adequate treatment recommendations for lipoma.<br>(ii) Potential treatment recommendations for degenerative/rheumatic changes are provided even though clinical symptoms (such as pain) that would necessitate treatment are not mentioned.<br>(iii) Weight loss, low-impact exercise, braces, canes, or walkers are recommended, which are of doubtful benefit in degenerative/rheumatic shoulder conditions.
	2	1. Manage glenohumeral degeneration, acromioclavicular arthritis, and bursitis conservatively, i.e. RICE protocol, physical therapy, pain management, and corticosteroid/hyaluronic acid injections. If conservative treatment fails, consider surgical intervention such as joint replacement (for glenohumeral degeneration) or arthroscopic debridement (for acromioclavicular arthritis and bursitis).<br>2. Similarly, manage the partially ruptured supraspinatus and subscapularis tendons and the tendinopathy of the long biceps tendon conservatively (as in 1.). If ineffective, consider surgical options such as arthroscopic rotator cuff repair.	(i) Adequate treatment recommendations, even though the scientific evidence of some recommended measures, such as hyaluronic acid injections, is doubtful.<br>(ii) Impingement syndrome per se does not justify the (contextually relatively aggressive) recommendation of total joint replacement. MR imaging-based assessment of glenohumeral cartilage status without clinical correlation does not justify this recommendation.

	3	1. Manage calcific tendinitis conservatively by addressing the pain and inflammation. Potential measures are rest, ice, pain management, physical therapy (to improve strength, flexibility, and range of motion), extracorporeal shock wave therapy, and corticosteroid injections. When conservative treatments are ineffective (rarely), consider surgical removal of the calcium deposits in rare cases.	(i) Adequate treatment recommendations.<br>(ii) Ultrasound-based needling of the calcium deposit is not mentioned even though widely performed.
	4	1. Conservative treatment (pain management, physical therapy)<br>2. Surgical options if conservative treatment fails to address degenerative shoulder changes	(i) Adequate treatment recommendations.
	5	1. Conservative treatment (pain management, physical therapy)<br>2. Surgical treatment if conservative treatment fails (arthroscopic rotator cuff repair, open rotator cuff repair, tendon transfer, reverse total shoulder replacement)	(i) Adequate treatment recommendations
	6	1. Conservative treatment for osteoarthritis (pain management, NSAIDs, physical therapy)<br>2. Close monitoring for joint effusion and osteochondromatosis<br>3. Surgical therapy if conservative measures fail (joint resurfacing, rotator cuff repair, joint replacement)	(i) Adequate treatment recommendations.<br>(ii) Recommendations are schematic; inverse shoulder prosthesis should be mentioned.
	7	1. Conservative treatment (pain management, anti-inflammatory medication, physical therapy), if successful: targeted physical therapy program<br>2. Re-assessment and imaging. If conservative treatment not successful consider surgical treatment (Bankart repair, bone graft)	(i) Adequate treatment recommendations.
	8	1. Conservative treatment (pain relief, anti-inflammatory medication, activity modification)<br>2. Injections of corticosteroids if conservative treatment is unsuccessful	(i)Adequate treatment recommendations.<br>(ii) Relation between pathologies not fully accounted for.
	9	1. Monitor swelling<br>2. If swelling causes discomfort: conservative treatment may be considered (physical therapy, pain management)<br>2. Follow-up imaging if swelling persists	(i) Adequate treatment recommendations.
	10	1. Conservative treatment (activity modification, cool shoulder, OTC pain relievers)<br>2. Physical therapy if symptoms do not improve<br>3. Corticosteroid injections if 1+2 do not provide sufficient relief<br>4. Surgical consultation if symptoms persist	(i) Adequate treatment recommendations.

Literature

1. Coakley G, Mathews C, Field M, Jones A, Kingsley G, Walker D, et al. BSR & BHPR, BOA, RCGP and BSAC guidelines for management of the hot swollen joint in adults. Rheumatology. 2006;45(8):1039-41.
2. Naziri Q, Beyer GA, Shah NV, Solow M, Hayden AJ, Nadarajah V, et al. Knee dislocation with popliteal artery disruption: A nationwide analysis from 2005 to 2013. Journal of Orthopaedics. 2018;15(3):837-41.
3. Balcarek P, Oberthür S, Hopfensitz S, Frosch S, Walde TA, Wachowski MM, et al. Which patellae are likely to redislocate? Knee Surgery, Sports Traumatology, Arthroscopy. 2014;22:2308-14.
4. Murphey MD, Choi JJ, Kransdorf MJ, Flemming DJ, Gannon FH. Imaging of osteochondroma: variants and complications with radiologic-pathologic correlation. Radiographics : a review publication of the Radiological Society of North America, Inc. 2000;20(5):1407-34.
